# Supplementary figures and images for: Systems Biomedicine of Rabies Delineates the Affected Signaling Pathways
Source: Front Microbiol. 2016 Nov 7;7:1688. doi: 10.3389/fmicb.2016.01688 (PMC5098112; doi:10.3389/fmicb.2016.01688)

# Degree Distribution

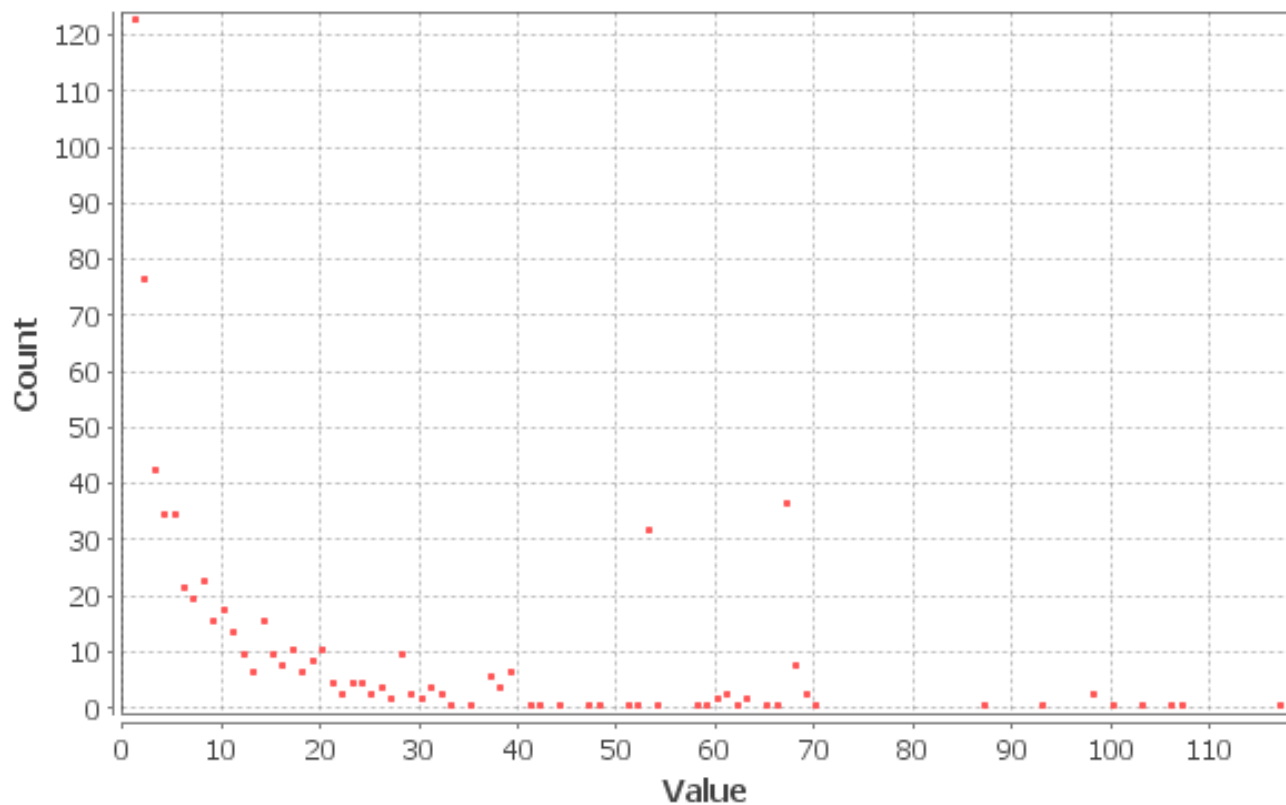

Supplement: Supplementary Figure 1 — The degree distribution of the refined SHIDEG-PPIN. [file Image1.pdf]
